# Early and Non-invasive Diagnosis of Aspergillosis Revealed by Infection Kinetics Monitored in a Rat Model

**DOI:** 10.3389/fmicb.2018.02356

**Published:** 2018-10-08

**Authors:** Anton Skriba, Tomas Pluhacek, Andrea Palyzova, Zbynek Novy, Karel Lemr, Marian Hajduch, Milos Petrik, Vladimir Havlicek

**Affiliations:** ^1^Institute of Microbiology of the Czech Academy of Sciences, Prague, Czechia; ^2^Department of Analytical Chemistry, Regional Centre of Advanced Technologies and Materials, Faculty of Science, Palacký University Olomouc, Olomouc, Czechia; ^3^Institute of Molecular and Translational Medicine, Faculty of Medicine and Dentistry, Palacký University Olomouc, Olomouc, Czechia

**Keywords:** liquid chromatography, mass spectrometry, PET, *Aspergillus fumigatus*, siderophores

## Abstract

**Background:** *Aspergillus fumigatus* is a ubiquitous saprophytic airborne fungus responsible for more than one million deaths every year. The siderophores of *A. fumigatus* represent important virulence factors that contribute to the microbiome-metabolome dialog in a host. From a diagnostic point of view, the monitoring of *Aspergillus* secondary metabolites in urine of a host is promising due to the non-invasiveness, rapidity, sensitivity, and potential for standardization.

**Methods:** Using a model of experimental aspergillosis in immunocompromised Lewis rats, the fungal siderophores ferricrocin (FC) and triacetylfusarinine C (TAFC) were monitored in rat urine before and after lung inoculation with *A. fumigatus* conidia. Molecular biomarkers in high-dose (HD) and low-dose (LD) infection models were separated using high performance liquid chromatography (HPLC) and were detected by mass spectrometry (MS). In the current work, we corroborated the *in vivo* MS infection kinetics data with micro-positron emission tomography/computed tomography (μPET/CT) kinetics utilizing ^68^Ga-labeled TAFC.

**Results:** In the HD model, the initial FC signal reflecting aspergillosis appeared as early as 4 h post-infection. The results from seven biological replicates showed exponentially increasing metabolite profiles over time. In *A. fumigatus*, TAFC was found to be a less produced biomarker that exhibited a kinetic profile identical to that of FC. The amount of siderophores contributed by the inoculating conidia was negligible and undetectable in the HD and LD models, respectively. In the μPET/CT scans, the first detectable signal in HD model was recorded 48 h post-infection. Regarding the MS assay, among nine biological replicates in the LD model, three animals did not develop any infection, while one animal experienced an exponential increase of metabolites and died on day 6 post-infection. All remaining animals had constant or random FC levels and exhibited few or no symptoms to the experiment termination. In the LD model, the TAFC concentration was not statistically significant, while the μPET/CT scan was positive as early as 6 days post-infection.

**Conclusion:** Siderophore detection in rat urine by MS represents an early and non-invasive tool for diagnosing aspergillosis caused by *A. fumigatus*. μPET/CT imaging further determines the infection location *in vivo* and allows the visualization of the infection progression over time.

## Introduction

*Aspergillus fumigatus* is a ubiquitous saprophytic airborne fungus that causes life-threatening pulmonary infections in immunocompromised hosts. *A. fumigatus* has also recently emerged as an important pathogen in immunocompetent patients in intensive care units. In 2018, David W. Denning reported that estimated 14,700,000 cases of aspergillosis occur resulting in 1,010,000 deaths every year^1^. On an annual basis, mainly invasive pulmonary aspergillosis (IPA; 200,000–400,000), chronic pulmonary aspergillosis (CPA; 1.5–3 M) and allergic bronchopulmonary aspergillosis (ABPA; 6–20 M) account for these aspergillosis cases, with mortality rates of 30–85%, 45% within 5 years, and <1%, respectively, for the treated cases of these three prevailing aspergillosis forms. These rapidly growing incidence numbers together with emerging pan-azole-resistant *Aspergillus* strains define aspergillosis as an important infectious disease for which no vaccine is yet available (Moore et al., 2017).

Methods routinely used in clinical practice to detect IPA, such as culture, serology, molecular, and radiology techniques, are often slow, invasive, and lack specificity or sensitivity (Luptakova et al., 2017). The identification of patients at high risk, appropriate prophylaxis, diagnostic surveillance, and early and reliable diagnostic tests remain important for improving patient management and underline the need for specific and sensitive diagnostic tools for the identification of IPA (Zhao et al., 2018). There are multiple as yet unvalidated molecular diagnostic tools, e.g., the detection of volatile compounds in the breath by gas chromatography and mass spectrometry (MS) (Koo et al., 2014) or proton nuclear magnetic resonance spectroscopy (Pappalardo et al., 2014). Newer molecular imaging and serology approaches have been identified, including the use of radiolabeled siderophores and antibodies, for positron emission tomography (Haas et al., 2015; Davies et al., 2017) and cytokine IL-8 detection (Goncalves et al., 2017b), respectively. All these techniques require rigorous examination as in our previous work dedicated to siderophore monitoring during *Aspergillus* infection (Pluhacek et al., 2016b). The validation of newly developed tools in a clinical setting represents a prerequisite for future inclusion in the *European Society of Clinical Microbiology and Infectious Diseases* guidelines (Ullmann et al., 2018) or the *European Organization for Research and Treatment of Cancer* criteria^2^.

Siderophores are low-molecular-weight iron-chelating molecules secreted by a diverse set of bacteria and fungi. Recent research has revealed that siderophores represent important microbial virulence factors that contribute to the microbiome-metabolome dialog in a host (Goncalves et al., 2017a), and the release of these factors is associated with nutrient procurement for microbial growth. After chelating iron, the uptake of ferri-siderophores is mediated by specific transporters, termed siderophore iron transporters (SITs) (Schrettl and Haas, 2011). Genomic investigation has revealed that SITs are encoded by all fungal species with genome sequences available. Consistently, non-siderophore-producing species have been shown to use SITs for the uptake of siderophores produced by other microorganisms. It is important to note that siderophore usage is confined to the fungal and bacterial kingdoms and that bacteria and mammals do not possess SIT-type transporters (Haas et al., 2008). Bacteria employ structurally different transporter types, e.g., ABC-transporters, for siderophore uptake (Schalk and Guillon, 2013). These differences among fungi, bacteria, and mammals significantly contribute to the specificity of an *in vivo* diagnostic strategy exploiting the siderophore system to improve disease diagnosis.

Further, siderophore detection in host specimens poses multiple advantages over other methods of aspergillosis detection (Savelieff and Pappalardo, 2017), including non-invasiveness, rapidity, sensitivity, and the potential for standardization upon careful quantitation. Conversely, metabolite-based methods do not allow the determination of infection location and require sample preparation and extraction, and the high performance liquid chromatography (HPLC)-MS/MS equipment is costly at the research stage of development. Although, the use of matrix-assisted laser desorption time-of-flight MS for the ribosomal typing of microbes involved in childhood diseases had to overcome the same obstacles a decade ago, now it is a common MS tool in many hospital microbiology departments (Havlicek et al., 2013).

There are several issues deserving further research in siderophore applications, such as specificity and the infection kinetics of biomarkers. TAFC and FC are considered panfungal markers because the production of these molecules by other microbial genera has been described (Pluhacek et al., 2016a; Ramirez-Garcia et al., 2018). This work is aimed to address the precise relationship between the *Aspergillus* siderophore level over time and the severity of infection in a rat model of pulmonary aspergillosis (Petrik et al., 2010).

## Materials and Methods

### Rat Pulmonary Infection Model

*Aspergillus fumigatus* 1059 CCF was obtained from the Culture Collection of Fungi, Faculty of Science, Charles University in Prague and was maintained on yeast medium slants (0.3% malt extract, 0.3% yeast extract, 0.5% peptone, and 0.5% glucose) at 4°C. To maintain a low siderophore content in the inoculation material, fungal conidia were harvested with Tween 80 in phosphate buffer, and the fungal hyphae were partly removed by filtration through 20 μm porosity Millipore paper (Merck Millipore, Prague, Czechia). The filtrate containing spores was then centrifuged, and wet conidia were resuspended in 0.05 M phosphate buffer with 0.1% Tween 80.

The progression of *A. fumigatus* infection was studied in a rat pulmonary infection model as described previously (Luptakova et al., 2017). Briefly, to induce neutropenia, the rats received repeated (5 days and 1 day before inoculation; 75 mg/kg) intraperitoneal injections of DNA-alkylating agent cyclophosphamide (Endoxan, Baxter, Prague, Czechia). To prevent possible bacterial superinfection, the animals were given antibiotics throughout the experiment. To minimize animal suffering, the introduction of *Aspergillus* spores into animals, ^68^Ga-tracer injection and small animal imaging were performed under 2% isoflurane anesthesia (Forane, Abbott Laboratories, Abbott Park, IL, United States). *A. fumigatus* infection was established by the intratracheal administration of *Aspergillus* spores at two different concentrations (1 × 10^4^ or 1 × 10^8^ CFU/mL). A 100 μL dose of *A. fumigatus* spores was administered using the TELE PACK VET X LED system equipped with a flexible endoscope (Karl Storz GmbH & Co., KG, Tuttlingen, Germany) under inhalation anesthesia. This homogenized extract (100 μL) was examined by HPLC-MS and served as the false-negative control for the siderophore signal derived solely from the inoculation. The three control animals (CTRL1, CTRL2, and CTRL3) underwent the same treatment as the infected animals, except for the administration of the *Aspergillus* spores.

For MS detection, the urine from experimental animals was collected twice a day starting 1 day before the inoculation until the end of the experiment. The animals treated with the high dose (HD) (1 × 10^8^ CFU/mL) of *Aspergillus* spores were sacrificed by exsanguination 2–3 days (depending on the infection progression) after the *Aspergillus* administration. The rats infected with the low dose (LD) of *Aspergillus* (1 × 10^4^ CFU/mL) were sacrificed by exsanguination 10 days after the inoculation. At the end of the experiment, serum samples and whole lungs were also collected.

All animal experiments were conducted in accordance with the regulations and guidelines of the Czech Animal Protection Act (No. 246/1992) and with the approval of the Ministry of Education, Youth and Sports of the Czech Republic (MSMT-21275/2016-2) and the institutional Animal Welfare Committee of the Faculty of Medicine and Dentistry of Palacký University in Olomouc. The care of research staff conformed to the general guidelines for the protection of the European Community (86/609/EEC, 200/54/EC 16) and included the use of respiratory protective equipment with the standard FFP2 equivalent to an N95 HEPA filter. The animal studies were performed using female 2- to 3-month-old Lewis rats (Envigo, Horst, Netherlands). For PET/CT, the number of animals was reduced to 3 for both (HD and LD) *in vivo* experiments. For MS metabolomics, we used 7 and 9 infected animals in the HD and LD models, respectively.

### Animal PET/CT Imaging

PET/CT images were acquired with an Albira PET/SPECT/CT small animal imaging system (Bruker Biospin Corporation, Woodbridge, CT, United States). Rats were intravenously (i.v.) injected with a ^68^Ga-radiolabeled tracer, prepared as described elsewhere (Petrik et al., 2012), at a dose of 5–10 MBq corresponding to 1–2 μg of TAFC per animal. Anesthetized animals were placed in a prone position in the Albira system before the start of imaging. Static PET/CT images were acquired over 40 min starting 45 min after the ^68^Ga-TAFC injection. A 10 min PET scan (axial field of view 148 mm) was performed, followed by a triple CT scan (axial field of view 65 mm, 45 kVp, and 400 μA with 400 projections). Scans were reconstructed with the Albira software (Bruker Biospin Corporation, Woodbridge, CT, United States) using the maximum likelihood expectation maximization (MLEM) and filtered backprojection (FBP) algorithms. After reconstruction, the acquired data were viewed and analyzed using the PMOD software (PMOD Technologies Ltd., Zürich, Switzerland). Three-dimensional volume-rendered images were obtained using the VolView software (Kitware, Clifton Park, NY, United States).

To follow the progression of *Aspergillus* infection over time, the animals were imaged at selected time points after the inoculation. The animals receiving a low *Aspergillus* dose (1 × 10^4^ CFU/mL) were scanned 3 h after the inoculation and then on days 2, 6, and 10 post-infection. The rats infected with the high (1 × 10^8^ CFU/mL) dose of *Aspergillus* spores were imaged at 3 h and on days 1, 2, and 3 post-infection. PET/CT images of the control animals were acquired at the beginning and at the end of the experiment (day 10).

### Chromatography and Mass Spectrometry

Mass spectrometry experiments were performed using 7 high dose (HD1–HD7), 9 low dose (LD1–LD9) and 3 control (CTRL1–CTRL3) animals (biological replicates) in the HD and LD models, respectively. The siderophore extractions from urine were performed according to a protocol adapted for metabolite profiling (Luptakova et al., 2017). Briefly, the urine samples (20 μL) were mixed with the FOX-MIX standard (EMC Microcollections GmbH, Tübingen, Germany; 10 μL; 1 μg/mL in 5% ACN, ferrioxamines B, D, G, and E), which served as an internal standard, and were diluted with methanol (110 μL) to provide a final methanolic solution of approximately 80%. The samples were gently shaken and incubated overnight at −80°C. The samples were then centrifuged at 14,000 ×*g* for 10 min (4–8°C), and the supernatant was transferred to a new 1.5 mL microcentrifuge tube, lyophilized to dryness and stored at −80°C.

Prior to HPLC/MS analysis in triplicate, all the samples were re-dissolved in 5% acetonitrile (60 μL). Each sample (5 μL) was loaded (20 μL loop) onto a BEH C18/1.7 μm, 2.1 mm × 5 mm VanGuard precolumn (Waters, Prague, Czechia) at a 50 μL/min flow rate. The siderophores were then separated on an analogous analytical column (1 × 100 mm) at 60°C with the following gradient: 0–1 min (2% B), 25 min (60% B), 28–33 min (95% B), and 34–45 min (2% B); where A and B represent 0.1% formic acid in water and 0.1% formic acid in acetonitrile, respectively.

The positive-ion ESI mass spectra were collected using a 12 T SolariX FTICR mass spectrometer (Bruker Daltonics, Billerica, MA, United States). The ion source was operated at 4,800 V with drying gas (N_2_) at a flow rate of 2.5 L/min. The collision voltage and time-of-flight values were −8.5 V and 1.2 ms, respectively. The mass spectra were collected in the CASI mode (700–1,000 *m/z* window) at an approximate 0.3 Hz frequency with a mass accuracy better than 3 ppm. The extracted ion chromatograms with 0.005 Da spectral width of Fe-ferricrocin (Fe-FC) and Fe-triacetylfusarinine C (Fe-TAFC) were used for the integration and adjusted to the response of Fe-ferrioxamine E (Fe-FOXE).

### Serology

Tests for 1,3-β-D-glucan (BDG) were performed using a Fungitell kit (Fungitell, Associates of Cape Cod, Falmouth, United States) with the rat serum and urine samples. The sample was considered positive if the cut-off value was >80 pg/mL.

### Statistical Analysis

The variability in the concentration of Fe-FC and Fe-TAFC within the technical and biological replicates was assessed by the coefficient of variation (**Supplementary Tables S3**, **S4**). The concentrations of Fe-FC and Fe-TAFC are presented as the mean ± standard error of mean (**Supplementary Table S4**). The diagnostic performance of LC-CASI-FTICR methodology was expressed as sensitivity and specificity for Fe-FC, and Fe-TAFC using the procedure described in literature (Trevethan, 2017; **Supplementary Table S1**). The statistical analyses were performed using Microsoft Excel 2016 (Microsoft Corporation, Redmond, WA, United States). Standard uptake values (SUVs) in the lungs of the imaged animals were calculated by PMOD software after the selection of corresponding region of interest (ROI) – the lungs.

## Results

The siderophores were quantified in the urine from infected rats using external matrix-matched calibration standards (EMC Microcollections GmbH, Germany). The calibration range for Fe-FC ranged from 0.05 to 10,000 ng/mL, which fully covered the concentration range in the actual samples (**Supplementary Figure S1**). The Fe-TAFC concentration in the urine from the infected animals was lower, and the calibration range was reduced accordingly (0.05–500 ng/mL).

### High-Dose (HD) Infection Model

**FIGURE 1:**
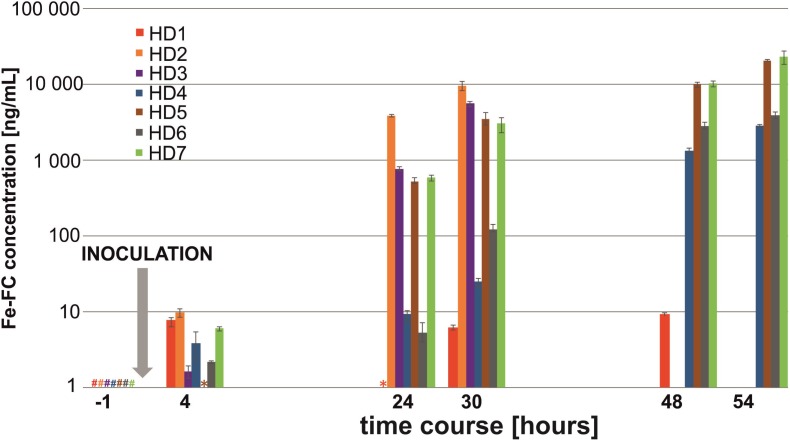
Infection diagram indicating the progression of aspergillosis in the HD rat model with seven biological replicates. Fe-FC concentration detected in rat urine is plotted on a logarithmic scale. A hash (#) denotes the Fe-FC concentration below limit of detection, while an asterisk (^∗^) denotes the Fe-FC concentration below limit of quantification. Missing bars at 48–54 h post-infection indicate animal death.

In the HD model, siderophore signals were clearly detected in six (HD1, HD2, HD3, HD4, HD6, and HD7) out of seven animals at 4 h post-infection (**Figure 1**). Analysis of the *A. fumigatus* inoculum revealed that negligible siderophore signal could potentially come from intra-cellular FC or extra-cellular TAFC, representing a potential background signal in this experiment. The maximum Fe-FC and Fe-TAFC false-positive signals were quantified as 322.5 and 34.2 pg, respectively, assuming that all siderophores in the inoculum (100 μL) were quantitatively secreted into the urine (0.217 μL) of an average animal.

An exponential increase in the siderophore signal was recorded 1 day post-infection, which indicated a severe *A. fumigatus* infection leading to prompt animal death. Out of seven animals, two (HD2, HD3) died during the experiment, one (HD1) or four (HD4–HD7) were sacrificed 48 or 54 h post-infection, respectively. A single immunocompromised animal resisted the infection with *Aspergillus*, revealing few aspergillosis symptoms and maintaining a constant urine Fe-FC concentration (HD1 animal in red, **Figure 1**). Interestingly, Fe-TAFC was found to be a biomarker produced less by the *A. fumigatus* CCF 1059 strain that exhibited an exponential profile identical to that of Fe-FC (**Supplementary Figure S2**). Moreover, we observed that urine samples from humans infected with *A. fumigatus* (data not reported here) have a siderophore ratio opposite to that of other *fumigati* strains. To evaluate the diagnostic strength of our highly selective LC-CASI-FTICR methodology, the sensitivity and specificity of the Fe-FC and Fe-TAFC screening were calculated for each data point of the HD kinetic experiments (**Supplementary Table S1**). The proposed LC-CASI-FTICR approach consistently provided 100% specificity and sensitivity for Fe-FC. In contrast, the sensitivity for Fe-TAFC gradually increased from 0 to 100% throughout the kinetics experiment, while the specificity remained unchanged at 100%.

**FIGURE 2:**
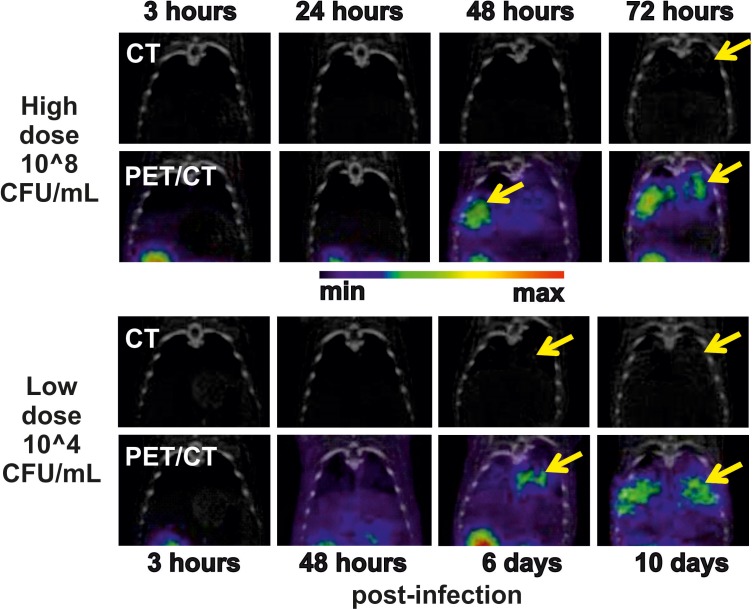
Thoracic coronal CT and PET/CT images of ^68^Ga-TAFC in infected (yellow arrow indicator) rats over time in experiments using high (top two rows) and low (bottom two rows) doses of conidia. The images in a row indicate the development of infection within a single animal.

In the μPET/CT scans, the first detectable radiodiagnostic signal was recorded 48 h post-infection (**Figure 2**). The presence of gray halo signs in the CT scans was apparent 72 h post-infection. SUVs in the lungs of the imaged HD animals were 0.21 ± 0.03 and 0.73 ± 0.10 percent of the injected dose per cubic centimeter (%ID/g) at the beginning and at the end of the experiment, respectively. No uptake in the lung region over time was detected in the non-infected rats in which the only visible organs were the following excretory organs: the kidneys, portions of the gastrointestinal tract and the bladder (**Supplementary Figure S3**). The SUVs in the lungs of the control animals were 0.21 ± 0.03 and 0.20 ± 0.03% ID/g at the beginning and at the end of the experiment, respectively.

Regarding the 1,3-β-D-glucan (BDG) testing, almost all the infected rat sera (HD2, HD4, HD5, HD6, and HD7) had BDG concentrations above the upper cut-off value (>617 pg/mL), indicating a severe panfungal infection (**Supplementary Figure S4**). Note that all the serum samples were collected at the end of the experiment. One-third of the control samples (CTRL1) yielded false-positive results. A better dynamic range for the analysis was documented with MS, which reflected the disease severity based on the siderophore profile. One HD animal (HD1) tolerated the infection more than the others as reflected both by BDG and MS testing (**Figure 1**).

### Low-Dose (LD) Infection Model

**FIGURE 3:**
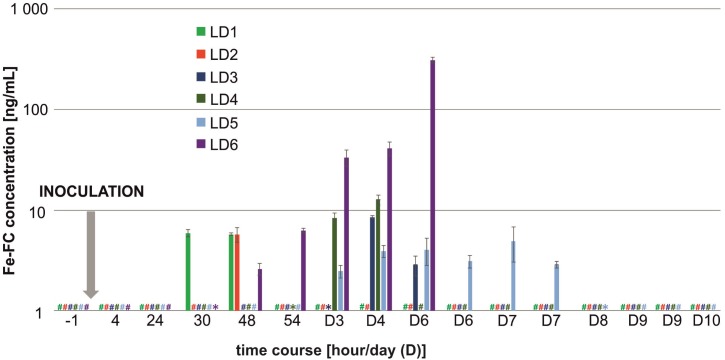
Fe-FC concentration profiles detected in rat urine in the LD model. A hash (#) denotes the Fe-FC concentration below the limit of detection, while an asterisk (^∗^) denotes the Fe-FC concentration below limit of quantitation. Missing bars at D6–D10 days post-infection indicate animal death. From days 6 to 9, two urine samples per day were collected. Rats LD7 – LD9 did not develop any signs of infection and no Fe-FC signal was detected throughout the LD experiment in their urine.

Among nine biological replicates in the LD model, three animals (LD7–LD9) did not show any metabolite production and did not develop any signs of infection. One animal (LD6) experienced an exponential increase in metabolites and died on day 6 post-infection (**Figure 3**). All five remaining animals (LD1–LD5) had constant or random FC levels and exhibited few or no symptoms to the end of the experiment (day 10). In the LD model, the TAFC concentration was not statistically significant, while the μPET/CT scan was positive as early as 6 days post-infection (**Figure 2**, bottom). For the LD-infected rats, the SUVs in the lungs of the imaged animals were 0.22 ± 0.02 and 0.38 ± 0.04% ID/g at the beginning and at the end of the experiment, respectively.

Interestingly, the infection foci recorded by μPET/CT increased to the end of the experiment, at which time the animals were without significant infection symptoms. We speculate that fungal mycelium in the lungs may have been able to take up additional ^68^Ga-labeled tracer independently of the invasion status of the pathogen cells (viable, dormant or dead). This interpretation agrees with the constant or even decreasing siderophore profiles in the urine as revealed by MS. The response of secondary metabolic processes to host defense mechanisms or possible antifungal treatment during fungal invasion thus remains unclear.

The BDG testing in urine was false-positive even for the time points preceding the actual inoculation of two selected rats (LD5 and HD9). Therefore, a comparison of the BDG results with those from MS was impossible (**Supplementary Table S2**). In comparison with MS, the testing of the rat sera for BDG further revealed two false-negative (LD1 and LD2) and two false-positive (LD4 and CTRL1) results. Good mutual agreement between the BDG and MS results was achieved in only the animal with the most highly developed infection (LD6) (**Supplementary Figure S4**).

## Conclusion

An accurate assessment of the invasive status of aspergillosis and the duration of the illness are required to determine whether siderophore levels correlate with early and/or late infection. In this work, a statistical analysis with adequate technical replication (**Supplementary Table S3**) revealed that the mean Fe-FC concentration in the urine samples of the rat cohort was 4.3 ± 1.3 ng/mL as early as 4 h post-infection in the HD model (**Supplementary Table S4**). The variability in biological replicates was expressed as the coefficient of variation (CV) and standard error of the mean (SEM). Fe-FC was observed to be a better tracer than Fe-TAFC for the *Aspergillus* strain used in our study. The signal originating from the inoculum itself was not significant. The concentrations of both the Fe-FC and Fe-TAFC metabolites in urine increased exponentially during the experiment (**Supplementary Figure S5**).

In the LD model, the signal derived from the inoculum was negligible, and the earliest detected siderophores appeared 30 h post-infection (**Figure 3**). More reliable results (for 1/3 of the animals, namely LD1, LD2, and LD6) were recorded 48 h post-infection and were reflected by the mean Fe-FC concentration of 4.7 ± 1.4 ng/mL. This observation indicates that siderophore detection by MS represents an extremely sensitive tool for early *Aspergillus* detection. Although we did not present sufficient statistics with the animal imaging, we showed that siderophore detection in the urine by MS was faster than the detection of infection by means of PET/CT. However, PET/CT imaging further determines the infection location *in vivo* and allows the visualization of the infection progression.

In the LD model, significant biological variability caused by different responses to the experimental aspergillosis was noted in the infection kinetics. A decline in siderophore production in the later stages of infection was detected, which may have been a response to increasing fungal burden and attenuating the immune system response (Savelieff and Pappalardo, 2017). In a recent study on experimental aspergillosis in mice, the first hyphae appeared after 12 h with an average length of 4.6 μm (Szigeti et al., 2018). The *ex vivo* histological examination showed that lung tissue lysis became visible 48 h post-infection and that the longest hyphae reached a length of 15 μm in infected animals 96 h post-infection.

Although many questions on the application of siderophores in *Aspergillus* detection remain to be answered, both PET and MS *in vivo* tools possess high potential for future human diagnostics. Further studies are needed, for example, to clarify the effect of antifungal prophylaxis on *Aspergillus* secondary metabolite production in human hosts. Moreover, a recent study (Carroll et al., 2016) reported that a subset of samples from patients with suspected invasive aspergillosis was TAFC-positive but galactomannan (GM)-negative. This observation suggested that TAFC is more sensitive than GM and that TAFC secretion may occur early during infection. We conclude that *Aspergillus* siderophore detection is non-invasive, rapid, sensitive, amenable to automation, and quantifiable and as such has great potential for standardization in human diagnostics.

## Author Contributions

AS performed the LC-MS experiments and prepared the figures. TP performed the LC-MS experiments and performed the statistical evaluation. AP prepared the inoculum and wrote the manuscript. ZN prepared radiolabeled siderophores and collected animal samples. KL wrote the manuscript. MH designed the animal imaging experiments and wrote the manuscript. MP produced the animal IPA model, collected the PET/CT data, and wrote the manuscript. VH conceived the experiments and wrote the manuscript.

## Conflict of Interest Statement

The authors declare that the research was conducted in the absence of any commercial or financial relationships that could be construed as a potential conflict of interest. The reviewer RK and handling Editor declared their shared affiliation.

## Footnotes

1 https://www.aspergillus.org.uk/slides/introduction-and-summary-global-disease-burden (access date April 21, 2018). Introductory opening talk by David W. Denning at 8th Advances against Aspergillosis international conference (February 1–3, 2018, Lisbon, Portugal).

2 http://www.eortc.org/guidelines/ (access date April 21, 2018).
