# Supplementary material for: Early and Non-invasive Diagnosis of Aspergillosis Revealed by Infection Kinetics Monitored in a Rat Model
Source: Front Microbiol. 2018 Oct 8;9:2356. doi: 10.3389/fmicb.2018.02356 (PMC6186828; doi:10.3389/fmicb.2018.02356)
Supplement: Supplementary file 1 [file Data_Sheet_1.pdf]

Supplementary Information

Early and non-invasive diagnosis of aspergillosis revealed by infection kinetics monitored in a rat model

Anton Skriba[1,$], Tomas Pluhacek[1,2,$], Andrea Palyzova[1], Zbynek Novy[3], Karel Lemr[1,2], Marian Hajduch[3], Milos Petrik[3,*],Vladimir Havlicek[1,2,*]

[1]Institute of Microbiology of the Czech Academy of Sciences, Prague 4, 142 20, Czechia
[2]Regional Centre of Advanced Technologies and Materials, Department of Analytical Chemistry, Olomouc, 771 46, Czechia
[3]Institute of Molecular and Translational Medicine, Faculty of Medicine and Dentistry, Palacky University, Olomouc, Czechia

[*]Corresponding authors, [$]Both authors contributed equally

Table of contents:

Figure S1: Calibration plots of Fe-FC (top) and Fe-TAFC (bottom) adjusted to the Fe-FOXE signal. Fe-FC concentration points were 0,05; 0,1, 0,5; 1; 5; 10; 50; 100; 500; 1,000; 5,000 and 1,000 ng/mL.

Figure S2: Fe-TAFC concentration detected in rat urine in the HD and LD infection models. Fe-TAFC concentration detected in rat urine is plotted in a logarithmic scale. A hash (#) denotes the Fe-TAFC concentration below the limit of detection, while an asterisk (*) denotes the Fe-TAFC concentration below limit of quantification. Missing bars at 48h – D6 post-infection indicate animal death.

Figure S3: Whole body three-dimensional volume-rendered PET/CT images of ^{68}Ga-TAFC in infected (yellow arrow indicates the infection) and non-infected rats. **(A)** PET/CT images of high-dose infected animals on the day of infection (on the left) and three days after inoculation (on the right). **(B)** PET/CT images of low-dose-infected animals on the day of infection (on the left) and ten days after inoculation (on the right). **(C)** PET/CT images of non-infected animals at the beginning (on the left) and at the end (on the right) of the experiment. S = site of injection, K = kidneys, G = gastrointestinal tract, U = urinary bladder.

Figure S4: Mutual comparison of siderophore (top) and 1,3-β-D-glucan (bottom) assays. Serum samples in the high-dose (HD1, HD2, HD4-HD7) low-dose (LD1-LD9) and control groups (CTRL1-CTRL3) (all collected at the end of the experiment) are represented by red, blue and orange colors, respectively. The BDG cut-off value defining positivity is 80 pg/mL, and the upper limit is 617 pg/mL. ND: not detected.

Figure S5: The mean Fe-FC concentrations detected in rat urine in the HD infection model.

Supplementary Table S1: The sensitivity and specificity of LC-CASI-FTMS determination of Fe-FC and Fe-TAFC kinetics in the high-dose model.

Supplementary Table S2: Urine BDG testing in rats LD5 and HD6 indicating false-positive results for the data points preceding the inoculation. Note: The BDG assay was not fully standardized for urine analysis.

Supplementary Table S3: Technical variability expressed as the coefficient of variation (CV) for each group of technical replications.

Supplementary Table S4: The variability in biological replicates expressed as the coefficient of variation (CV) in the high-dose-infected animals. SEM: the standard error of the mean.

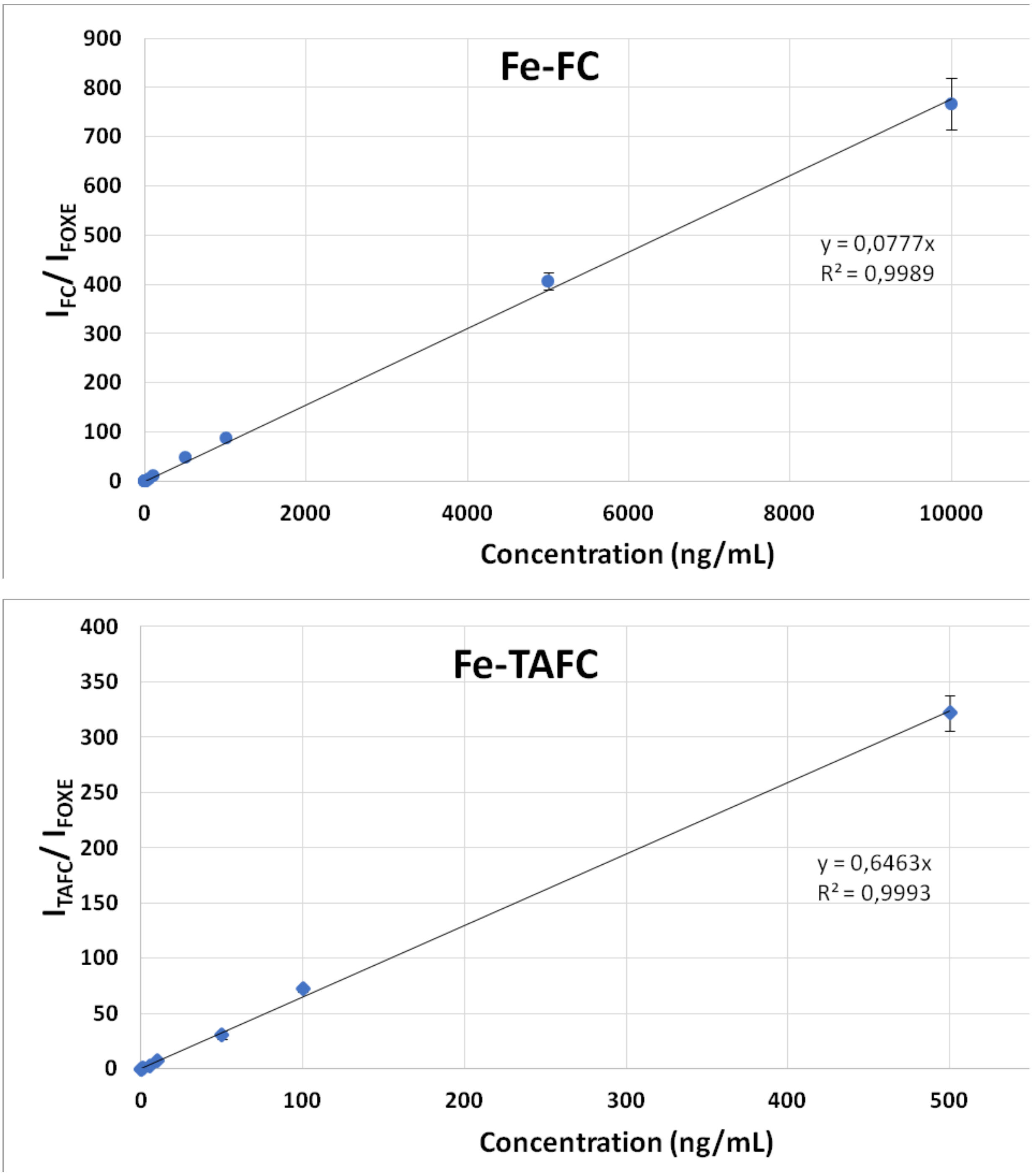

Figure S1: Calibration plots of Fe-FC (top) and Fe-TAFC (bottom) adjusted to the Fe-FOXE signal. Fe-FC concentration points were 0,05; 0,1, 0,5; 1; 5; 10; 50; 100; 500; 1,000; 5,000 and 1,000 ng/mL.

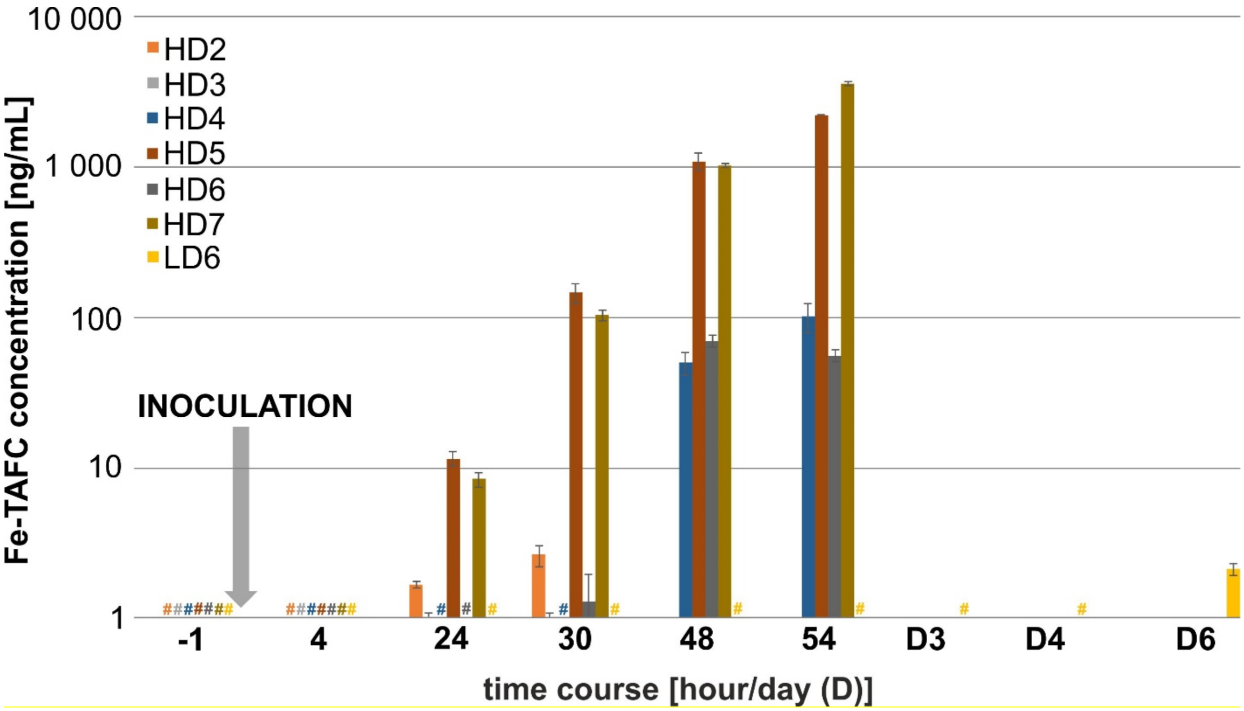

Figure S2: Fe-TAFC concentration detected in rat urine in the HD and LD infection models. Fe-TAFC concentration detected in rat urine is plotted in a logarithmic scale. A hash (#) denotes the Fe-TAFC concentration below the limit of detection, while an asterisk (*) denotes the Fe-TAFC concentration below limit of quantification. Missing bars at 48h – D6 post-infection indicate animal death.

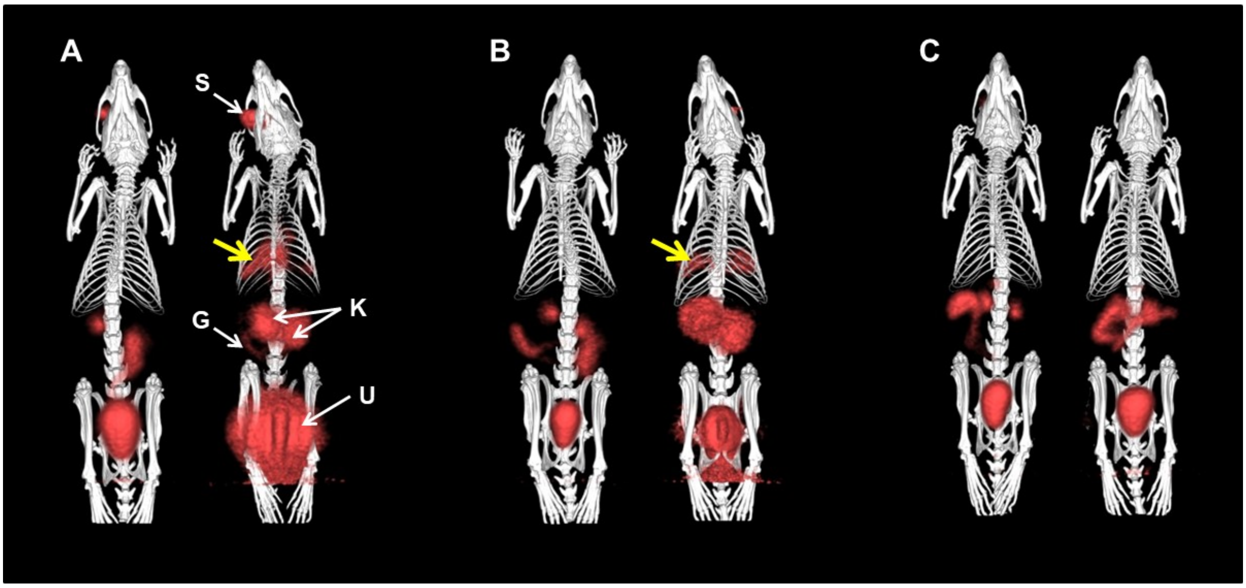

Figure S3: Whole body three-dimensional volume-rendered PET/CT images of ^{68}Ga-TAFC in infected (yellow arrow indicates the infection) and non-infected rats. **(A)** PET/CT images of high-dose-infected animals on the day of infection (on the left) and three days after inoculation (on the right). **(B)** PET/CT images of low-dose-infected animals on the day of infection (on the left) and ten days after inoculation (on the right). **(C)** PET/CT images of non-infected animals at the beginning (on the left) and at the end (on the right) of the experiment. S = site of injection, K = kidneys, G = gastrointestinal tract, U = urinary bladder.

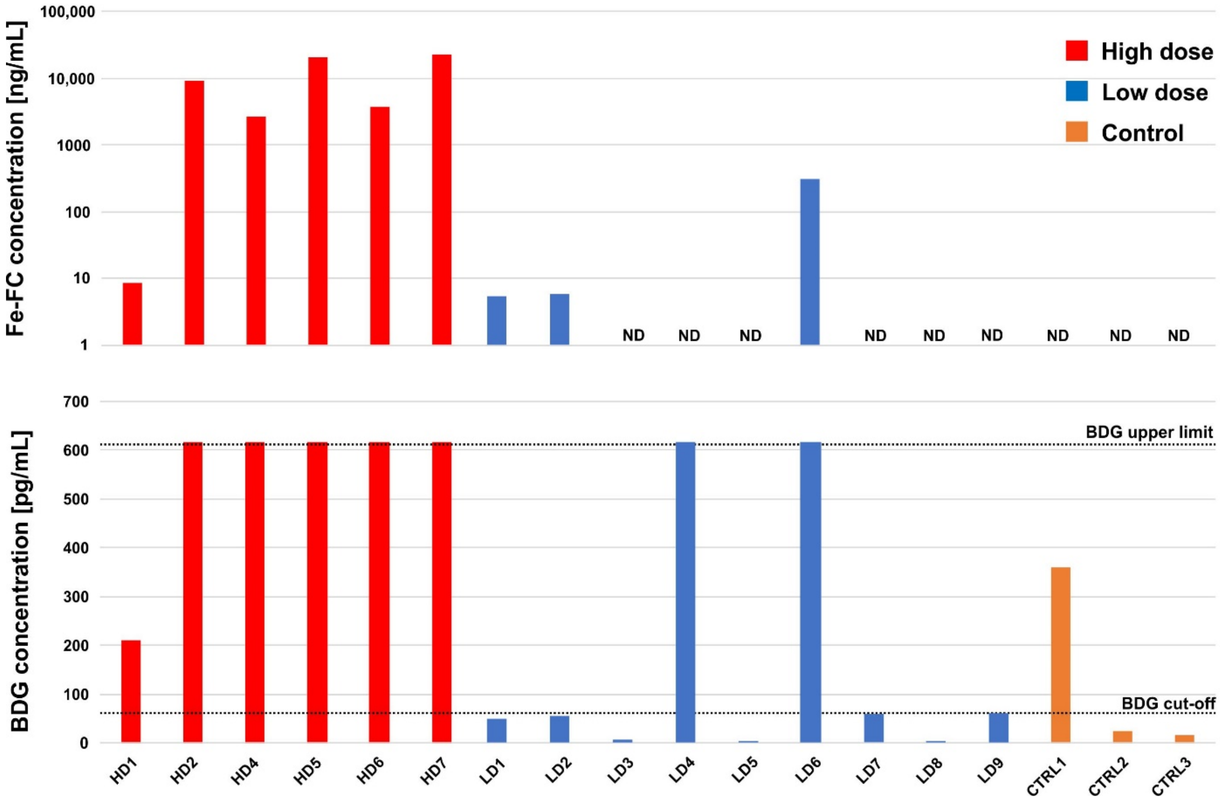

Figure S4: Mutual comparison of siderophore (top) and 1,3-β-D-glucan (bottom) assays. Serum samples in the high-dose (HD1, HD2, HD4-HD7) low-dose (LD1-LD9) and control groups (CTRL1-CTRL3) (all collected at the end of the experiment) are represented by red, blue and orange colors, respectively. The BDG cut-off value defining positivity is 80 pg/mL, and the upper limit is 617 pg/mL. ND: not detected.

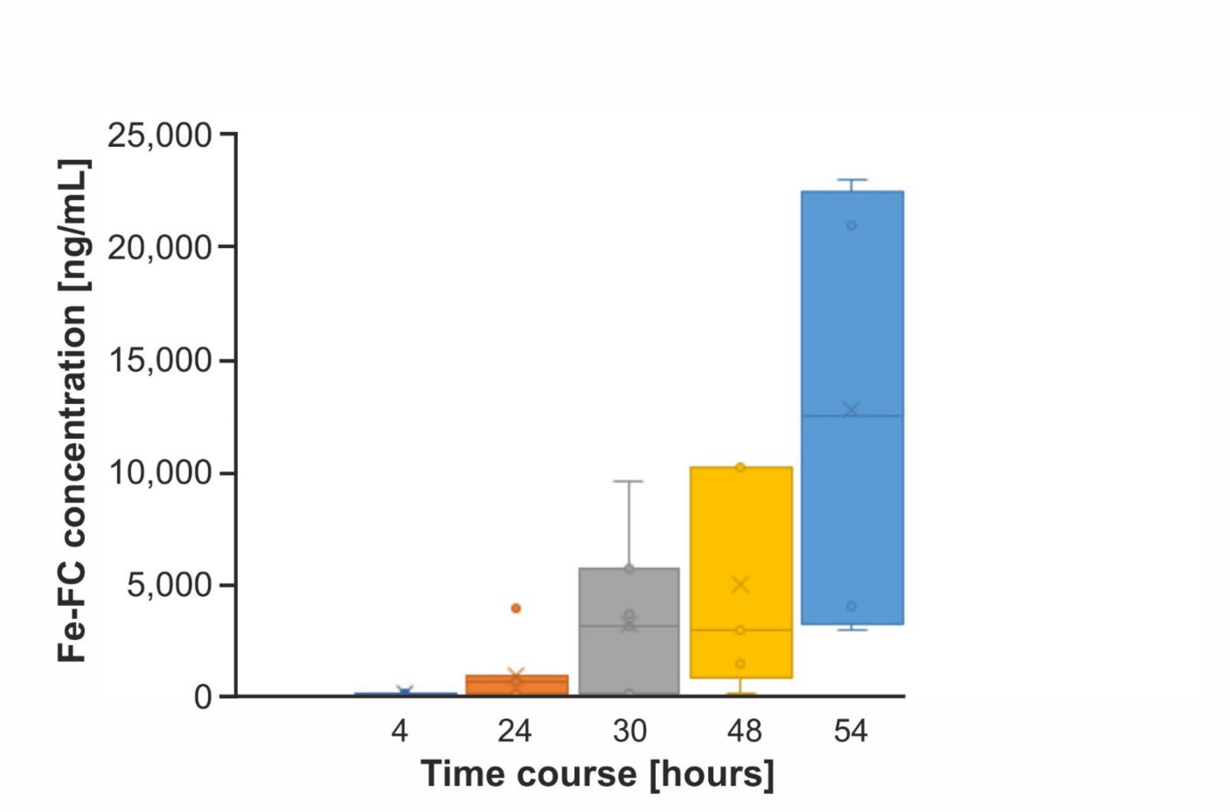

Figure S5: The mean Fe-FC concentrations detected in rat urine in the HD infection model.

Supplementary Table S1: The sensitivity and specificity of LC-CASI-FTMS determination of Fe-FC and Fe-TAFC kinetics in the high-dose model.

Time after inoculation	Fe-FC		Fe-TAFC	
	Sensitivity (%)	Specificity (%)	Sensitivity (%)	Specificity (%)
4 h	100 (7/7)	100 (3/3)	0 (0/7)	100 (3/3)
24 h	100 (7/7)	100 (3/3)	57 (4/7)	100 (3/3)
30 h	100 (7/7)	100 (3/3)	71 (5/7)	100 (3/3)
48 h	100 (5/5)	100 (3/3)	80 (4/5)	100 (3/3)
54 h	100 (4/4)	100 (3/3)	100 (4/4)	100 (3/3)

Supplementary Table S2: Urine BDG testing in rats LD5 new and HD6 indicating false-positive results for the data points preceding the inoculation. Note: The BDG assay was not fully standardized for urine analysis.

LD5		HD6	
Data point	pg/mL	Data point	pg/mL
-25 h	>617.2	-25 h	>617.2
-19 h	>617.2	- 19 h	>617.2
-1 h	>617.2	-1 h	>617.2
4 h	420.3	4 h	>617.2
24 h	>617.2	24 h	>617.2
30 h	>617.2	30 h	>617.2
48 h	478.4	48 h	>617.2
54 h	>617.2	54 h	>617.2
D3	>617.2		
D4	>617.2		
D6	>617.2		
D6	>617.2		
D7	>617.2		
D7	>617.2		
D8	>617.2		
D8	>617.2		
D9	236.5		
D9	>617.2		
D10	>617.2		

Supplementary Table S3: Technical variability expressed as the coefficient of variation (CV) for each group of technical replications.

	Fe-FC	Fe-TAFC
**Low dose**	0.047±0.010	0.071±0.033
**High dose**	0.051±0.007	0.065±0.016

Supplementary Table S4: The variability in biological replicates expressed as the coefficient of variation (CV) in the high-dose-infected animals. SEM: the standard error of the mean.

Time after inoculation	Siderophore	Mean (ng/mL)	±SEM (ng/mL)	CV
4 h	Fe-FC	4.3	1.3	0.783
	Fe-TAFC	0.0	0.0	NAN
24 h	Fe-FC	794.5	500.6	1.667
	Fe-TAFC	3.1	1.8	1.493
30 h	Fe-FC	3 019.4	1 296.3	1.136
	Fe-TAFC	36.0	23.1	1.698
48 h	Fe-FC	4 743.1	2 134.0	1.006
	Fe-TAFC	442.7	247.5	1.250
54 h	Fe-FC	12 284.5	5 239.4	0.853
	Fe-TAFC	1 470.1	849.0	1.155
